# Supplementary material for: Analysis of the optimal patterns of serum alpha fetoprotein (AFP), AFP-L3% and protein induced by vitamin K absence or antagonist-II (PIVKA-II) detection in the diagnosis of liver cancers
Source: PeerJ. 2025 Jul 21;13:e19712. doi: 10.7717/peerj.19712 (PMC12288744; doi:10.7717/peerj.19712)
Supplement: Supplemental Information 1 [file peerj-13-19712-s001.docx]

**Table S1 *P*-values of comparisons for AUC of tumor markers and diagnostic models in diagnosing HCC and** **non-HCC^#♦^.**

| **Marker/model** | **GAP-TALAD** | **GALAD** | **C-GALAD** | **ASAP** | **GALAD-C** | **GAAP** | **C-GALAD Ⅱ** | **PIVKA-Ⅱ** | **AFP** | **AFP-L3%** |
| --- | --- | --- | --- | --- | --- | --- | --- | --- | --- | --- |
| GAP-TALAD | - | - | - | - | - | - | - | - | - | - |
| GALAD | 0.2440 | - | - | - | - | - | - | - | - | - |
| C-GALAD | 0.1377 | 0.4936 | - | - | - | - | - | - | - | - |
| ASAP | 0.0854 | 0.2868 | 0.2201 | - | - | - | - | - | - | - |
| GALAD-C | 0.0524 | 0.1291 | 0.4199 | 0.7807 | - | - | - | - | - | - |
| GAAP | 0.0433^*^ | 0.0855 | 0.2619 | 0.5004 | 0.3504 | - | - | - | - | - |
| C-GALAD-Ⅱ | 0.0044^*^ | 0.0214^*^ | 0.1216 | 0.1658 | 0.1298 | 0.1616 | - | - | - | - |
| PIVKA-Ⅱ | < 0.0001^*^ | 0.0005^*^ | < 0.0001^*^ | < 0.0001^*^ | 0.0004^*^ | 0.0005^*^ | 0.2430 | - | - | - |
| AFP | 0.0001^*^ | 0.0001^*^ | 0.0018^*^ | 0.0031^*^ | 0.0053^*^ | 0.0068^*^ | 0.1941 | 0.9946 | - | - |
| AFP-L3% | < 0.0001^*^ | < 0.0001^*^ | < 0.0001^*^ | < 0.0001^*^ | < 0.0001^*^ | < 0.0001^*^ | 0.0102^*^ | 0.2198 | 0.0556 | - |

**Notes.**

^#^Comparisons were conducted by DeLong tests.

^♦^Non-HCC: CCA and benign liver diseases.

^*^Significant difference.

AUC: area under receiver operating characteristic (ROC) curve; HCC: hepatocellular carcinoma; PIVKA-Ⅱ: protein induced by vitamin K absence or antagonist-Ⅱ; AFP: alpha fetoprotein; AFP-L3%: percentage of AFP-L3 (culinaris agglutinin strong binding) to total AFP; CCA: cholangiocarcinoma.
