# Supplementary material for: Analysis of the optimal patterns of serum alpha fetoprotein (AFP), AFP-L3% and protein induced by vitamin K absence or antagonist-II (PIVKA-II) detection in the diagnosis of liver cancers
Source: PeerJ. 2025 Jul 21;13:e19712. doi: 10.7717/peerj.19712 (PMC12288744; doi:10.7717/peerj.19712)
Supplement: Supplemental Information 2 [file peerj-13-19712-s002.docx]

**Table S2 *P*-values of comparisons for AUC of tumor markers and diagnostic models in diagnosing liver cancers (HCC and CCA)^#♦^.**

| **Marker/model** | **GALAD-C** | **GAAP** | **GALAD** | **ASAP** | **C-GALAD** | **C-GALAD Ⅱ** | **PIVKA-Ⅱ** | **AFP-L3%** | **CEA** | **CA19-9** |
| --- | --- | --- | --- | --- | --- | --- | --- | --- | --- | --- |
| GALAD-C | - | - | - | - | - | - | - | - | - | - |
| GAAP | 0.5993 | - | - | - | - | - | - | - | - | - |
| GALAD | 0.3786 | 0.4498 | - | - | - | - | - | - | - | - |
| ASAP | 0.2837 | 0.3113 | 0.9238 | - | - | - | - | - | - | - |
| C-GALAD | 0.2134 | 0.2392 | 0.6303 | 0.3713 | - | - | - | - | - | - |
| C-GALAD Ⅱ | 0.0001^*^ | 0.0002^*^ | 0.0004^*^ | 0.0030^*^ | 0.0065^*^ | - | - | - | - | - |
| PIVKA-Ⅱ | < 0.0001^*^ | < 0.0001^*^ | 0.0002^*^ | < 0.0001^*^ | < 0.0001^*^ | 0.5047 | - | - | - | - |
| AFP-L3% | < 0.0001^*^ | < 0.0001^*^ | < 0.0001^*^ | < 0.0001^*^ | < 0.0001^*^ | 0.0131^*^ | 0.0742 | - | - | - |
| CEA | < 0.0001^*^ | < 0.0001^*^ | < 0.0001^*^ | < 0.0001^*^ | < 0.0001^*^ | 0.0001^*^ | 0.0013^*^ | 0.0372^*^ | - | - |
| CA19-9 | < 0.0001^*^ | < 0.0001^*^ | < 0.0001^*^ | < 0.0001^*^ | < 0.0001^*^ | 0.0001^*^ | 0.0005^*^ | 0.0109^*^ | 0.8119 | - |

**Notes.**

^#^Comparisons were conducted by DeLong tests.

^♦^Diagnosing liver cancers (HCC and CCA) and benign liver diseases.

^*^Significant difference.

AUC: area under receiver operating characteristic (ROC) curve; HCC: hepatocellular carcinoma; CCA: cholangiocarcinoma; PIVKA-Ⅱ: protein induced by vitamin K absence or antagonist-Ⅱ; AFP: alpha fetoprotein; AFP-L3%: percentage of AFP-L3 (culinaris agglutinin strong binding) to total AFP; CEA: carcinoembryonic antigen; CA19-9: carbohydrate antigen 19-9.
