# Supplementary material for: Analysis of the optimal patterns of serum alpha fetoprotein (AFP), AFP-L3% and protein induced by vitamin K absence or antagonist-II (PIVKA-II) detection in the diagnosis of liver cancers
Source: PeerJ. 2025 Jul 21;13:e19712. doi: 10.7717/peerj.19712 (PMC12288744; doi:10.7717/peerj.19712)
Supplement: Supplemental Information 3 [file peerj-13-19712-s003.docx]

**Table S3 *P*-values of comparisons for AUC of tumor markers and diagnostic models in diagnosing early liver cancers and benign liver diseases^#♦^.**

| **Marker/model** | **GALAD** | **GALAD-C** | **GAAP** | **C-GALAD** | **ASAP** | **C-GALAD Ⅱ** | **PIVKA-Ⅱ** | **AFP-L3%** | **CA19-9** | **CEA** |
| --- | --- | --- | --- | --- | --- | --- | --- | --- | --- | --- |
| GALAD | - | - | - | - | - | - | - | - | - | - |
| GALAD-C | 0.5931 | - | - | - | - | - | - | - | - | - |
| GAAP | 0.6416 | 0.9244 | - | - | - | - | - | - | - | - |
| C-GALAD | 0.3447 | 0.6032 | 0.8701 | - | - | - | - | - | - | - |
| ASAP | 0.2926 | 0.6278 | 0.7847 | 0.8578 | - | - | - | - | - | - |
| C-GALAD Ⅱ | 0.0310^*^ | 0.0553 | 0.0053^*^ | 0.1075 | 0.1223 | - | - | - | - | - |
| PIVKA-Ⅱ | 0.0005^*^ | 0.0007^*^ | 0.0001^*^ | 0.0023^*^ | 0.0039^*^ | 0.0574 | - | - | - | - |
| AFP-L3% | 0.0001^*^ | 0.0001^*^ | < 0.0001^*^ | 0.0002^*^ | 0.0004^*^ | 0.0150^*^ | 0.9865 | - | - | - |
| CA19-9 | 0.0001^*^ | 0.0001^*^ | 0.0001^*^ | 0.0005^*^ | 0.0007^*^ | 0.0178^*^ | 0.4706 | 0.4783 | - | - |
| CEA | < 0.0001^*^ | < 0.0001^*^ | < 0.0001^*^ | < 0.0001^*^ | < 0.0001^*^ | 0.0028^*^ | 0.2967 | 0.2950 | 0.7149 | - |

**Notes.**

^#^Comparisons were conducted by DeLong tests.

^♦^Early liver cancers: HCC with BCLC stages 0 to A and CCA with TNM stages 0 to Ⅱ.

^*^Significant difference.

AUC: area under receiver operating characteristic (ROC) curve; PIVKA-Ⅱ: protein induced by vitamin K absence or antagonist-Ⅱ; AFP: alpha fetoprotein; AFP-L3%: percentage of AFP-L3 (culinaris agglutinin strong binding) to total AFP; CA19-9: carbohydrate antigen 19-9; CEA: carcinoembryonic antigen; HCC: hepatocellular carcinoma; BCLC: Barcelona clinic liver cancer; CCA: cholangiocarcinoma; TNM: primary tumor, regional lymph nodes and distant metastasis.
